# Interpretation of acid–base metabolism on arterial blood gas samples via machine learning algorithms

**DOI:** 10.1007/s11845-024-03767-6

**Published:** 2024-08-01

**Authors:** Habib Ozdemir, Muhammed Ikbal Sasmaz, Ramazan Guven, Akkan Avci

**Affiliations:** 1 Health Data Research and Artificial Intelligence Applications Institute, Health Institutes of Turkiye, Istanbul, Türkiye; 2 Faculty of Medicine, Department of Emergency Medicine, Manisa Celal Bayar University, Manisa, Türkiye; 3 Department of Emergency Medicine, Istanbul Basaksehir Cam and Sakura City Research and Training Hospital, Health Science University, Istanbul, Türkiye; 4 Department of Emergency Medicine, Adana City Research and Training Hospital, Health Science University, Adana, 01060 Türkiye

**Keywords:** Acid–base metabolism, Arterial blood gas, Artificial Intelligence, Machine learning

## Abstract

**Background:**

Arterial blood gas evaluation is crucial for critically ill patients, as it provides essential information about acid–base metabolism and respiratory balance, but evaluation can be complex and time-consuming. Artificial intelligence can perform tasks that require human intelligence, and it is revolutionizing healthcare through technological advancements.

**Aim:**

This study aims to assess arterial blood gas evaluation using artificial intelligence algorithms.

**Methods:**

The study included 21.541 retrospective arterial blood gas samples, categorized into 15 different classes by experts for evaluating acid–base metabolism status. Six machine learning algorithms were utilized; accuracy, balanced accuracy, sensitivity, specificity, precision, and F1 values of the models were determined; and ROC curves were drawn to assess areas under the curve for each class. Evaluation of which sample was estimated in which class was conducted using the confusion matrices of the models.

**Results:**

The bagging classifier (BC) model achieved the highest balanced accuracy with 99.24%, whereas the XGBoost model reached the highest accuracy with 99.66%. The BC model shows 100% sensitivity for nine classes and 100% specificity for 10 classes, and the model correctly predicted 6438 of 6463 test samples and achieved an accuracy of 99.61%, with an area under the curve > 0.9 in all classes on a class basis.

**Conclusion:**

The machine learning models developed exhibited remarkable accuracy, sensitivity, and specificity in predicting the status of acid–base metabolism. However, implementing these models can aid clinicians, freeing up their time for more intricate tasks.

## Introduction

Artificial intelligence (AI) is the ability of a computer or computer-controlled robot to perform tasks that require human intelligence and judgment, often done by humans [1]. Artificial intelligence is gradually changing the perspective of research in the field of healthcare and biomedicine, and scientific and public awareness in the field of artificial intelligence has emerged thanks to recent technological developments [2]. Nowadays, artificial intelligence systems use machine learning methods by performing complex calculations to identify patterns from data, unlike first-generation artificial intelligence systems that depend on medical knowledge and strong decision rules that have been formulated by experts. Machine learning is a subset of artificial intelligence that focuses on the use of data and algorithms by imitating the way humans learn and can gradually improve its accuracy. Machine learning algorithms are mainly divided into four categories according to the problem to be solved: supervised learning, unsupervised learning, semi-supervised learning, and reinforcement learning [3]. Supervised learning is a type of machine learning where a computer program learns to identify patterns and relationships between inputs and outputs by studying labeled examples. The program uses this labeled data to build a model that can accurately predict the output for new inputs. The main purpose of supervised learning is to achieve specific goals by training the program on a task-oriented approach [4]. The most common supervised tasks are “classification,” which separates the data, and “regression,” which fits the data. The use of machine learning methods allows for the creation of artificial intelligence (AI) applications that aid in the detection of previously unidentified patterns within data without the need for the specification of decision rules for each specific task or the consideration of complex interactions between input features. Therefore, machine learning has emerged as the preferred framework for the development of AI utilities [2].

Assessment of acid–base balance is a crucial component in the management of critically ill patients. Arterial blood gas analysis is utilized to evaluate both the acid–base and respiratory balance through the measurement of key parameters such as pH, bicarbonate levels, partial oxygen pressure (PaO_2_), the partial pressure of carbon dioxide (PaCO_2_), and oxygen saturation (SaO_2_) [5]. Recent advancements in point-of-care testing (POCT) analyzers have facilitated the measurement of glucose, electrolyte levels, kidney function, bilirubin, and hemoglobin levels in arterial blood gas analysis. In essence, arterial blood gas analysis is utilized for the purposes of diagnosing and monitoring metabolic and respiratory acidosis and alkalosis, identifying the type of respiratory failure, evaluating the efficacy of the treatment given, necessity and monitoring oxygen therapy, and determining sudden-onset and unexplained dyspnea [6]. Timely identification of these disorders through blood gas analysis is crucial for the clinician who must act quickly in emergency room and intensive care unit settings. Accurate interpretation of arterial blood gas analysis is paramount in the diagnosis and treatment of patients, as it is a commonly used laboratory parameter. Specifically, some physicians may encounter challenges in distinguishing between compensation and mixed disorder as a response to the primary disorder resulting from the disease. Therefore, in order to ensure a systematic and accurate interpretation of arterial blood gas analysis, certain methods are proposed [7–9]. The purpose of employing these methods in the evaluation process is to assess the results step by step and determine whether there is an appropriate response or an additional pathology present.

This study aims to predict the acid–base metabolism status of the patient using artificial intelligence methods and arterial blood gas sample results.

## Methods

### Data source and collection

The study was conducted at the Celal Bayar University Faculty of Medicine Hospital, which is a tertiary care university hospital. Blood gas samples were collected by educated hospital blood drawing personnel from inpatients and outpatients into the lithium heparinized syringes. Appropriate samples were analyzed, and inappropriate samples (clotted samples, insufficient samples, etc.) were rejected by laboratory personnel. The study included the results of 21.541 arterial blood gas samples obtained from 2.668 adult patients admitted to our hospital between January 2022 and July 2022. Arterial blood gas samples were analyzed using RAPIDLAB 500e and RAPIDLAB 1200 analyzers (Siemens Healthineers, Erlangen, Germany). Patient age, gender, and arterial blood gas result parameters including pH, PaCO_2_ (the partial pressure of carbon dioxide), PaO_2_ (oxygen pressure), sodium, potassium, chloride, ionized calcium, glucose, lactate, FO_2_Hb (oxygenated hemoglobin fraction), FCOHb (carboxyhemoglobin fraction), FHHb (deoxygenated hemoglobin fraction), FMetHb (methemoglobin fraction), total hemoglobin, Hct (hematocrit), osmolarity, SaO_2_ (oxygen saturation), ctCO_2_ (CO_2_ concentration), HCO_3_^−^ (bicarbonate concentration), base excess, and BEecf (extracellular fluid base excess) were obtained retrospectively from the laboratory information management system.

### The determination of acid–base metabolism disorders

Acid–base disorders are defined by changes in serum pH, the partial pressure of carbon dioxide (PaCO_2_), and serum bicarbonate (HCO_3_^−^) levels. The normal range of blood pH is between 7.35 and 7.45, the normal range of PaCO_2_ is 35–45 mmHg, and the acceptable range of HCO_3_^−^ is 22–26 mmol/L.

The arterial blood gas results of the cases were interpreted by an emergency medicine specialist. Arterial blood gas analysis was evaluated in five steps following current guidelines recommended in emergency medicine practice [9, 10].

First of all, the pH is evaluated, and its normal range is between 7.35 and 7.45. Values below 7.35 indicate acidosis, whereas values above 7.45 indicate alkalosis. Maintaining the homeostasis of blood pH requires coordination between the respiratory and renal systems. However, the body also employs a series of buffer systems that resist changes in blood pH. A pH lower than 6.8 or higher than 7.8 is incompatible with life [11].

After evaluation of the pH, partial pressure of carbon dioxide (PaCO_2_) and bicarbonate (HCO_3_^−^) levels are evaluated. In acid–base disorders, the arterial partial pressure of carbon dioxide (PaCO_2_), which is an indicator of alveolar ventilation, is examined for the evaluation of the respiratory component. For the evaluation of the metabolic component, the bicarbonate (HCO_3_^−^) level is examined. In practice, when an acid–base disorder is detected, the PaCO_2_ level is evaluated first. If the pH and PaCO_2_ levels change in the same direction, it is considered that the primary disorder is metabolic in origin; if they change in the opposite direction, it is considered that the primary disorder is respiratory in origin. Then, the serum HCO_3_^−^ level is evaluated.

In the metabolic disorders, the PaCO_2_ response is evaluated to assess sub-types of the metabolic acid–base disorders. For this purpose, Winter’s formula is used to calculate the expected PaCO_2_ level in the presence of metabolic acidosis (Winter’s formula: Expected PaCO_2_ = 1.5* HCO_3_^−^ + 8 (± 2)). To calculate the expected PaCO_2_ level in the presence of metabolic alkalosis, the following formula is used: Expected PaCO_2_ = 40 + 0.6*Δ HCO_3_^−^ (ΔHCO_3_^−^ = the difference between the patient’s HCO_3_^−^ level and the normal HCO_3_^−^ level (24 mmol/L)). If the patient’s PaCO_2_ level is at the expected PaCO_2_ range, it is considered respiratory compensation that occurs in metabolic acidosis or alkalosis.

For metabolic acidosis situations, in partially compensated metabolic acidosis, the PaCO_2_ level is higher than the expected PaCO_2_ level but less than 35 mmHg; in isolated metabolic acidosis, PaCO_2_ is within the normal range; in combined metabolic and respiratory acidosis, PaCO_2_ is above 45 mmHg.

In the presence of metabolic alkalosis, in partially respiratory compensated metabolic alkalosis, the PaCO_2_ level is lower than the expected PaCO_2_ level but higher than 45 mmHg; in isolated metabolic alkalosis, PaCO_2_ is within the normal range; in metabolic and respiratory alkalosis, PaCO_2_ is lower than 35 mmHg.

For respiratory disorders, the expected pH is calculated to evaluate the metabolic component. In the presence of respiratory acidosis, the effect of the respiratory component (PaCO_2_) on pH is calculated using the following formula: ΔpH = 0.008*(PaCO_2_ − 40). In acute respiratory acidosis, the patient’s pH level is around the calculated pH value; in partially compensated respiratory acidosis, the patient’s pH level is above the calculated pH value; and in respiratory and metabolic acidosis, the patient’s pH level is below the calculated pH value. In the presence of respiratory alkalosis, the effect of the respiratory component (PaCO_2_) on pH is calculated using the following formula: ΔpH = 0.008*(40 − PaCO_2_). In acute respiratory alkalosis, the patient’s pH level is close to the calculated pH value; in partially metabolic compensated respiratory alkalosis, the patient’s pH level is below the calculated pH value; and in respiratory and metabolic alkalosis, the patient’s pH level is above the calculated pH value.

Even if the pH is within the normal range, the levels of PaCO_2_ and HCO_3_^−^ are evaluated. In some acid–base disorders, even though the pH value is normal, the levels of PaCO_2_ and HCO_3_^−^ may be low or high. In this case, a mixed disorder may occur where both metabolic acidosis and respiratory alkalosis or metabolic alkalosis and respiratory acidosis are present together.

After the evaluation, the results of patients’ arterial blood gas analyses were labeled into 15 different categories: isolated metabolic acidosis (IMAc), metabolic acidosis with partial respiratory response (MAcPRR), metabolic acidosis with respiratory response (MAcRR), isolated respiratory acidosis (IRAc), respiratory acidosis with partial metabolic response (RAcPMR), isolated metabolic alkalosis (IMAl), metabolic alkalosis with partial respiratory response (MAlPRR), metabolic alkalosis with respiratory response (MAlRR), isolated respiratory alkalosis (IRAl), respiratory alkalosis with partial metabolic response (RAlPMR), normal, metabolic acidosis, and respiratory acidosis (MAcRAc), metabolic acidosis and respiratory alkalosis (MAcRAl), metabolic alkalosis and respiratory alkalosis (MAlRAl), and metabolic alkalosis and respiratory acidosis (MAlRAc). Arterial blood gas results for each sample were labeled as belonging to a single class.

### Data preprocessing

Preprocessing of the data is the method of deleting or replacing data from a data collection that is incomplete, noisy, or inconsistent. It is a crucial phase in the development of any prediction model. There is no universal preprocessing phase for all types of data since data sets differ from one another [12].

After collecting the data, the data preprocessing stage was carried out to make it suitable for the training of machine learning models. In the data preprocessing stage, arterial blood gas parameters were selected as input variables. The missing data in the dataset was imputed using one of the most common imputation methods, the k-nearest neighbor (kNN) method, because some machine learning models that we used do not work with missing data [13, 14] and the number of missing data and the percentages given in a supplement. To ensure that continuous variables contribute equally to the model, all variables were normalized to the [0,1] range using the scaling method in Python [15]. To prevent overfitting of the models, we checked whether there were samples with the same values in all blood gas feature parameters.

### Machine learning algorithms

The dataset was split into two different datasets for training and testing purposes: a training dataset and a test dataset. For the training dataset, 70% of the data was used, while the remaining 30% was used for testing. The training and test datasets were stratified to include an equal proportion of acid–base metabolism classes. To determine the optimal model parameters, the training dataset was further split into training and validation datasets, and hyperparameters were tuned. After validation, the models were trained on the training set using the optimal parameters for each model. The trained models were then used to predict the acid–base metabolism status of previously unseen samples in the test dataset. The development, training, and testing of the machine learning models were carried out on the Python 3.11 platform. Appropriate classification machine algorithms were used in the study, and classification was performed using the bagging classifier (BC) [15], XGBoost [16], artificial neural networks (ANN) [15], CatBoost [17], random forest (RF) [15], and logistic regression (LR) [15] machine learning algorithms.

The total, training, and test number of samples for each class are provided in Table 1.

**Table 1:** Number of total, training, and testing samples for acid–base status classes

Acid–base metabolism status	Total samples	Training samples	Test samples
Normal	5.217	3.652	1.565
Isolated metabolic alkalosis	4.004	2.803	1201
Isolated respiratory alkalosis	2.539	1.777	762
Metabolic acidosis and respiratory alkalosis	1.747	1.223	524
Metabolic alkalosis and respiratory acidosis	1.738	1.217	521
Metabolic alkalosis and respiratory alkalosis	1.589	1.112	477
Metabolic alkalosis with partial respiratory response	991	694	297
Isolated metabolic acidosis	841	589	252
Respiratory alkalosis with partial metabolic response	765	535	230
Respiratory acidosis with partial metabolic response	614	430	184
Isolated respiratory acidosis	505	353	152
Metabolic acidosis and respiratory acidosis	497	348	149
Metabolic acidosis with respiratory response	266	186	80
Metabolic acidosis with partial respiratory response	215	150	65
Metabolic alkalosis with respiratory response	13	9	4
Total	21.541	15.078	6.463

### Statistical analysis and performance evaluation

Statistical analysis and performance evaluation were conducted to assess the performance of the machine learning models. The Kolmogorov–Smirnov test was used to test whether the data followed a normal distribution. The model outputs were obtained using the Python platform, and the performance was evaluated using accuracy, balanced accuracy, precision, and F1 score values. For each model, class-specific receiver operating characteristic (ROC) curves were plotted, and the area under the curve (AUC) was calculated, considering sensitivity and specificity values to determine the performance of the model for the respective class [18]. A confusion matrix was used to investigate how many patients were predicted correctly or incorrectly in each class by the models. SHAP (SHapley Additive exPlanations) values were obtained and graphically displayed to interpret how the selected model made predictions and to analyze how the arterial blood gas parameters affected the acid–base metabolism status and to what extent. The trained model was used to calculate the SHAP values and determine the contribution of each arterial blood gas parameter to predict the acid–base metabolism status for the examples in the test dataset [19].

## Results

### Dataset features

A total of 2.668 patients were included in the study, of whom 1.542 (57.80%) were male and 1.126 (42.20%) were female. The mean and standard deviation age values of all patients, males, and females were found to be 62.37 (± 15.55), 60.75 (± 15.56), and 64.91 (± 15.20), respectively. It was seen that the blood gas parameters used as features in the model training did not follow a normal distribution, and the median, 25th percentile, and 75th percentile values are presented in Table 2.

**Table 2:** Median, 25th percentile, and 75th percentile values of the arterial blood gas samples

	Median	25th percentile	75th percentile
pH	7.45	7.39	7.5
PaCO_2_ (mmHg)	37.7	33	43.4
HCO_3_^−^ (mmol/L)	25.2	22.2	28.9
ctCO_2_ (mmol/L)	26.3	23.3	30.2
PaO_2_ (mmHg)	93.9	69.5	138
SaO_2_ (%)	97.1	93.9	98.5
tHb (g/dL)	10.8	9.7	12.2
Hct (%)	32	29	36
FO_2_Hb (%)	96.1	92.7	97.6
FCOHb (%)	0.5	0.3	1
FHHb (%)	2.9	1.5	6.1
FMetHb (%)	0.3	0.2	0.5
Na (mmol/L)	136.5	133.3	139.9
K (mmol/L)	3.86	3.51	4.23
Cl (mmol/L)	102	98	106
Ionized calcium (mmol/L)	1.04	0.98	1.1
ABE (mmol/L)	1.3	-1.9	5
Anion Gap (mmol/L)	12.5	9.8	15.6
BEecf (mmol/L)	1.1	-2.3	5.3
Glucose (mg/dL)	145	112	195
Lactate (mmol/L)	1.46	1.03	2.31
Osm (mmol/kg)	281.7	274.6	289.5

### Performance evaluation of models

The obtained dataset was used to train and test six different models for predicting acid–base metabolism status. The accuracy and balanced accuracy values for BC, XGBoost, CatBoost, RF, ANN, and logistic regression algorithms are shown comparatively in Table 3.

**Table 3:** Accuracy and balanced accuracy values of the models

Model	Accuracy	Balanced accuracy
Bagging classifier	0.996132	**0.992487**
XGBoost	**0.996596**	0.988702
CatBoost	0.995358	0.93838
RF	0.984063	0.939035
ANN	0.993501	0.916778
Logistic regression	0.931301	0.879132

According to Table 3, when looking at the accuracy values, XGBoost, BC, CatBoost, RF, ANN, and logistic regression models are ranked from highest to lowest, respectively. For balanced accuracy values, the ranking is BC, XGBoost, ANN, CatBoost, RF, and logistic regression. Because BC has the highest balanced accuracy value, it was selected as the best model (Table 4).

**Table 4:** Sensitivity, specificity, precision, and F1 scores of the bagging classifier model based on acid–base metabolism

Bagging classifier	Sensitivity	Specificity	Precision	F1 score
Isolated metabolic acidosis	100%	100%	100%	100%
Metabolic acidosis with partial respiratory response	95.38%	100%	100%	97.64%
Metabolic acidosis with respiratory response	100%	99.89%	91.95%	95.81%
Isolated respiratory acidosis	100%	100%	100%	100%
Respiratory acidosis with partial metabolic response	99.46%	100%	100%	99.73%
Isolated metabolic alkalosis	100%	100%	100%	100%
Metabolic alkalosis with partial respiratory response	100%	100%	100%	100%
Metabolic alkalosis with respiratory response	100%	99.98%	80.00%	88.89%
Isolated respiratory alkalosis	100%	100%	100%	100%
Respiratory alkalosis with partial metabolic response	96.52%	99.87%	96.52%	96.52%
Normal	100%	100%	100%	100%
Metabolic acidosis and respiratory acidosis	100%	99.98%	99.33%	99.67%
Metabolic acidosis and respiratory alkalosis	99.24%	100%	100%	99.62%
Metabolic alkalosis and respiratory alkalosis	98.32%	99.87%	98.32%	98.32%
Metabolic alkalosis and respiratory acidosis	99.81%	100%	100%	99.90%

### Evaluation of the confusion matrix

Confusion matrices for each class of the BC model are provided in Table 5. The model misclassified 25 out of 6.463 test samples, with an accuracy of 99.61% and a balanced accuracy of 99.25%.

**Table 5:** Confusion matrix for bagging classifier

Predicted															
Truth	IMAc	MAcPRR	MAcRR	IRAc	RAcPMR	IMAl	MAlPRR	MAlRR	IRAl	RAlPMR	Normal	MAcRAc	MAcRAl	MAlRAl	MAlRAc
IMAc	252	-	-	-	-	-	-	-	-	-	-	-	-	-	-
MAcPRR	-	62	3	-	-	-	-	-	-	-	-	-	-	-	-
MAcRR	-	-	80	-	-	-	-	-	-	-	-	-	-	-	-
IRAc	-	-	-	152	-	-	-	-	-	-	-	-	-	-	-
RAcPMR	-	-	-	-	183	-	-	-	-	-	-	1	-	-	-
IMAl	-	-	-	-	-	1201	-	-	-	-	-	-	-	-	-
MAlPRR	-	-	-	-	-	-	297	-	-	-	-	-	-	-	-
MAlRR	-	-	-	-	-	-	-	4	-	-	-	-	-	-	-
IRAl	-	-	-	-	-	-	-	-	762	-	-	-	-	-	-
RAlPMR	-	-	-	-	-	-	-	-	-	222	-	-	-	8	-
Normal	-	-	-	-	-	-	-	-	-	-	1565	-	-	-	-
MAcRAc	-	-	-	-	-	-	-	-	-	-	-	149	-	-	-
MAcRAl	-	-	4	-	-	-	-	-	-	-	-	-	520	-	-
MAlRAl	-	-	-	-	-	-	-	-	-	8	-	-	-	469	-
MAlRAc	-	-	-	-	-	-	-	1	-	-	-	-	-	-	520

The model correctly predicted all the samples in the isolated metabolic acidosis, metabolic acidosis with respiratory response, isolated respiratory acidosis, isolated metabolic alkalosis, metabolic alkalosis with partial respiratory response, metabolic alkalosis with respiratory response, isolated respiratory alkalosis, normal, and metabolic acidosis, and respiratory acidosis classes.

### The ROC curves and AUC values

**Fig. 1:**
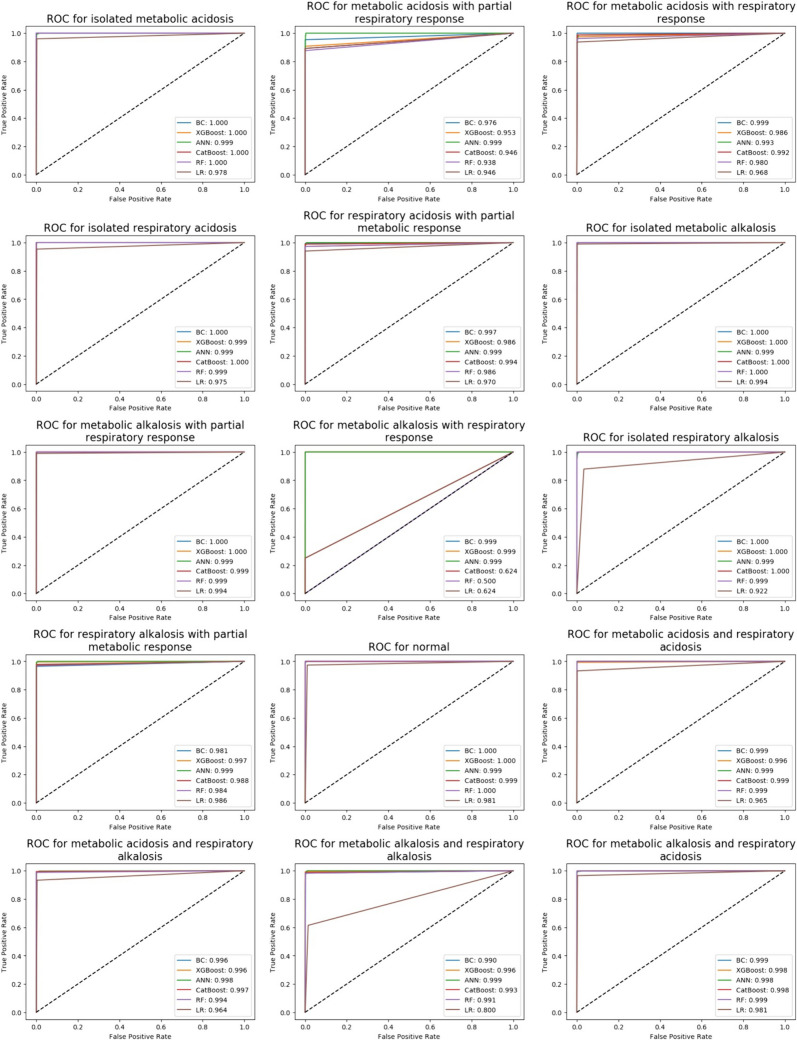
ROC curves of models based on acid–base metabolism status

In order to compare the predictive performance of each algorithm, ROC curves and area under the curve (AUC) values for acid–base metabolism were presented in Fig. 1.

### The contribution of features to the model

**Fig. 2:**
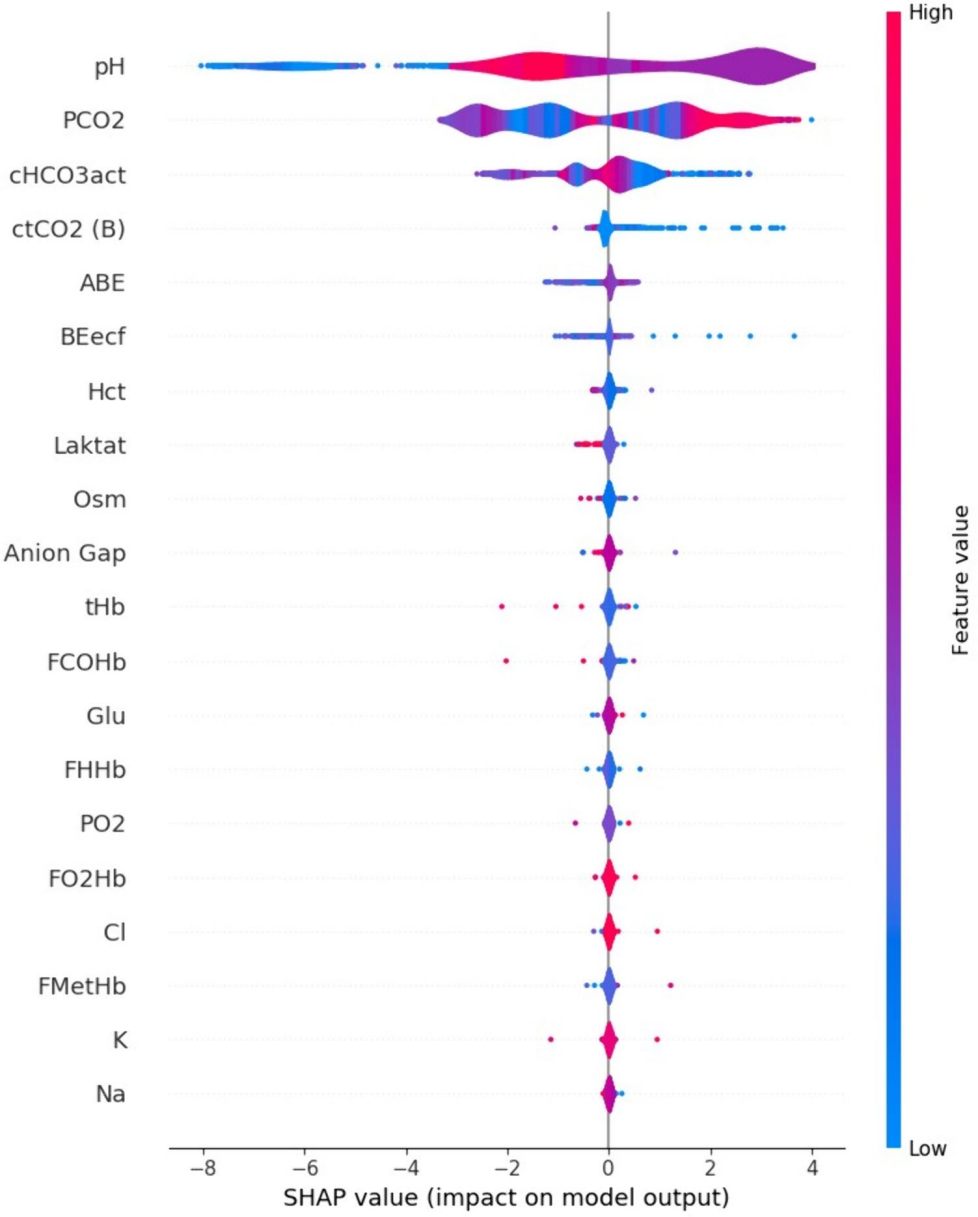
The contribution of features to the bagging classifier model

The SHAP values of arterial blood gas parameters used to predict the acid–base metabolism status in the BC model are shown in Fig. 2. When examining the contribution of features to the model, it can be observed that pH, PaCO_2_, and actual HCO_3_^−^ concentration have the highest contribution values in predicting the acid–base metabolism status via a machine learning algorithm.

## Discussion

Arterial blood gas (ABG) analysis is one of the most important laboratory methods that provide reliable information about the metabolic and respiratory status of patients. Evaluating a patient’s acid–base balance is crucial in critical patient management. Interpreting blood gas results requires time-consuming and experience-requiring mathematical calculations. Especially for clinicians racing against time in emergency rooms and intensive care units, using blood gas analysis to detect these disorders is essential for diagnosis and treatment.

The use of structured health data in electronic health records with machine learning and decision support systems is expanding. In particular, clinical decision support algorithms are rapidly increasing in the diagnosis field due to their quick, accurate, and inexpensive nature [20].

The study aims to predict the evaluation of blood gas analysis using machine learning models with classification models. The performance of the classification models was evaluated using accuracy, balanced accuracy, sensitivity, specificity, precision, F1 score, and ROC curves.

XGBoost and BC algorithms have shown the highest performance in evaluating blood gas results with machine learning models. While the XGBoost algorithm has the highest accuracy value (99.66%), the BC model has the highest balanced accuracy value (99.24%). The lowest performance was observed in the LR model, with accuracy and balanced accuracy values of 93.13% and 87.91%, respectively. In the test dataset consisting of 6.364 samples, 25 patients were predicted incorrectly by the BC algorithm, while 22 patients were wrongly predicted by the XGBoost algorithm. However, because of the imbalance in the number of samples among the classes based on acid–base metabolism in the dataset, the balanced accuracy metric was used to select the best-performing algorithm, and the BC algorithm was considered as the best model.

All models achieved AUC values above 0.9 for all classes, except for the metabolic alkalosis and respiratory alkalosis class, where the LR algorithm showed excellent performance with a value of 0.80 and for the metabolic alkalosis with respiratory response class; AUC values below 0.7 were obtained for CatBoost, RF, and LR algorithms, indicating insufficient discrimination. The lower performance of the LR model in the metabolic alkalosis and respiratory alkalosis class may be due to other machine learning models being more advanced than the LR model. The low number of samples in the metabolic alkalosis with respiratory response class may also result in low AUC values.

According to the balanced accuracy metric, the BC model that showed the best performance had AUC values of 1.00 in the isolated metabolic acidosis, isolated respiratory acidosis, isolated metabolic alkalosis, metabolic alkalosis with partial respiratory response, isolated respiratory alkalosis, and normal classes. The AUC values were 0.999 in the metabolic acidosis with respiratory response, metabolic alkalosis with respiratory response, metabolic acidosis and respiratory acidosis, and metabolic alkalosis and respiratory acidosis classes; 0.997 in the respiratory acidosis with partial metabolic response class; 0.996 in the metabolic acidosis and respiratory alkalosis class; 0.990 in the metabolic alkalosis and respiratory alkalosis class; and 0.981 in the respiratory alkalosis with partial metabolic response class. The AUC value in the metabolic acidosis with partial respiratory response class was 0.976.

The model accurately predicted all samples in the isolated metabolic acidosis, isolated respiratory acidosis, isolated metabolic alkalosis, metabolic alkalosis with partial respiratory response, isolated respiratory alkalosis, and normal classes without error, and its sensitivity and specificity values were determined to be 100%.

The model correctly predicted 62 out of 65 samples in the metabolic acidosis with partial respiratory response class, with a sensitivity of 95.38% and a specificity of 100%.

In the metabolic acidosis with respiratory response class, all 80 samples were correctly predicted with a sensitivity of 100%, and the specificity was determined to be 99.89% because the model misclassifies three patients from the metabolic acidosis with partial respiratory response class and four patients from the metabolic acidosis and respiratory alkalosis class as metabolic acidosis with respiratory response.

For the respiratory acidosis with partial metabolic response class, the model correctly predicted 183 out of 184 samples, resulting in a sensitivity of 99.46% and a specificity of 100%.

In the metabolic alkalosis with respiratory response class, the model correctly predicted all four samples, resulting in a sensitivity of 100%. The model misclassified one patient from the metabolic alkalosis and respiratory acidosis class as metabolic alkalosis with respiratory response, resulting in a specificity of 99.98%.

For the respiratory alkalosis with partial metabolic response class, the model correctly predicted 222 out of 230 samples, resulting in a sensitivity of 96.52%. The model misclassified eight patients from the metabolic alkalosis and respiratory alkalosis class as respiratory alkalosis with partial metabolic response, resulting in a specificity of 99.87%.

In the metabolic acidosis and respiratory acidosis class, the model correctly predicted all 149 patients, resulting in a sensitivity of 100%. The model misclassified one patient from respiratory acidosis with partial metabolic response class as metabolic acidosis and respiratory acidosis, resulting in a specificity of 99.98%.

In the metabolic acidosis and respiratory alkalosis class, the model correctly predicted 520 out of 524 samples, resulting in a sensitivity of 99.24% and a specificity of 100%.

For the metabolic alkalosis and respiratory alkalosis class, the model correctly predicted 469 out of 477 samples, resulting in a sensitivity of 98.32%. The model misclassified eight patients from the respiratory alkalosis with partial metabolic response class as metabolic alkalosis and respiratory alkalosis, resulting in a specificity of 99.87%.

The model accurately predicted 520 out of 521 samples in the class of metabolic alkalosis and respiratory acidosis, resulting in a sensitivity of 99.81% and specificity of 100%.

During the interpretation of blood gas results in the test dataset, the model made errors in predicting 25 samples (0.35%). However, the model correctly predicted the main acid–base disorder in the misclassified samples but made errors in interpreting the metabolic or respiratory response based solely on the acid–base disorder, resulting in incorrect classification.

In our study, machine learning methods were used to evaluate arterial blood gas results for 15 different classes. The strengths of the study include the inclusion of 21.541 arterial blood gas samples requested and processed in our clinical laboratory, the training of models with 15.078 samples, and the prediction of blood gas evaluations with 6.463 samples using the trained models. The use of a large number of sample data is believed to have contributed to the accuracy, balanced accuracy, and predictive power of the models.

Kajanan et al. conducted a study in which machine learning methods were utilized to predict respiratory failure using arterial blood gas results of 700 patients, utilizing pH, PaCO_2_, PaO_2_, HCO_3_^−^, and SaO_2_ tests. They achieved an average accuracy of 98.45% with the XGBoost model [12]. In our study, all parameters of the reported arterial blood gas results were evaluated by the model, allowing for the differentiation of various types of respiratory failure, as well as the identification of partial, complete, or mixed compensatory disorders with high performance using the developed model. Additionally, the models were trained with a large number of samples.

The study conducted by Rodríguez-Villar et al. involved real-time analysis of ABG samples for bedside testing. The main objective of the research was to clinically assess the algorithm for acid–base interpretation in comparison to senior experienced clinicians in a clinical setting. According to the findings, the algorithm demonstrated a sensitivity of 90.0%, a specificity of 97.2%, and an overall accuracy of 95.9% in predicting acid–base status [21]. In the study conducted by Rodríguez-Villar et al., the algorithm they developed is a math-based algorithm, and machine learning methods were not used. In our study, machine learning methods were used to predict acid–base status, and our global accuracy value was found to be 99.66% for the XGBoost model.

Zare et al. proposed a dataset consisting of 313 elements, 172 of which were mandatory and 141 were optional, to assist in the evaluation of arterial blood gas values in artificial intelligence-based systems [22]. In our study, while age and gender data were obtained from the hospital information management system, machine learning models were fed with 22 arterial blood gas parameters, trained, and predictions were made. Although the model was trained without the features having any weight, thanks to machine learning, the most important features were determined to be pH, PaCO_2_, and HCO_3_^−^ seen in descending order of importance, as used in blood gas evaluation. With more comprehensive datasets, it is possible to make predictions for outcomes such as mortality, sepsis, and acute kidney injury using artificial intelligence.

## Conclusion

The study found that XGBoost performed the best in terms of accuracy, whereas the BC model showed balanced accuracy and achieved a value of 99.25%. The developed machine learning models were able to predict the acid–base metabolism status with high accuracy, sensitivity, and specificity. Assessing the acid–base metabolism status requires complex calculations, which can be a time-consuming process for clinicians. However, the developed models can assist clinicians and allow them to focus on more complex tasks.

## Data Availability

Data and materials are reachable from hospital automation information systems.
